# Supplementary material for: Gliding toward an understanding of the origin of flight in bats
Source: PeerJ. 2024 Jul 25;12:e17824. doi: 10.7717/peerj.17824 (PMC11283779; doi:10.7717/peerj.17824)
Supplement: Supplemental Information 3 [file peerj-12-17824-s003.pdf]

Supplementary Information for
Gliding towards an understanding of the origin of flight in bats

This PDF file includes:

Supplementary Results

Figure S1: PCA with fossil and extant species of interest labeled.

Table S1: Loadings for forelimb and hind limb PCAs with extant mammals and extinct bats.

Table S2: Statistical results for OUM model fitting analyses on the extant-only dataset.

Table S3: Optima and bootstrapping results for best-supported OUM model.

Table S4: Statistical results for OUM model fitting analyses on the extant+extinct dataset.

Table S5: Optima values for the best-fitting OUM model results for extant+extinct dataset.

Table S6: Fits of multivariate evolutionary models to extant+extinct dataset.

Other supplementary materials for this manuscript

Data File S1: Morphological dataset for 231 mammal species and 4 extinct bats (.csv file).

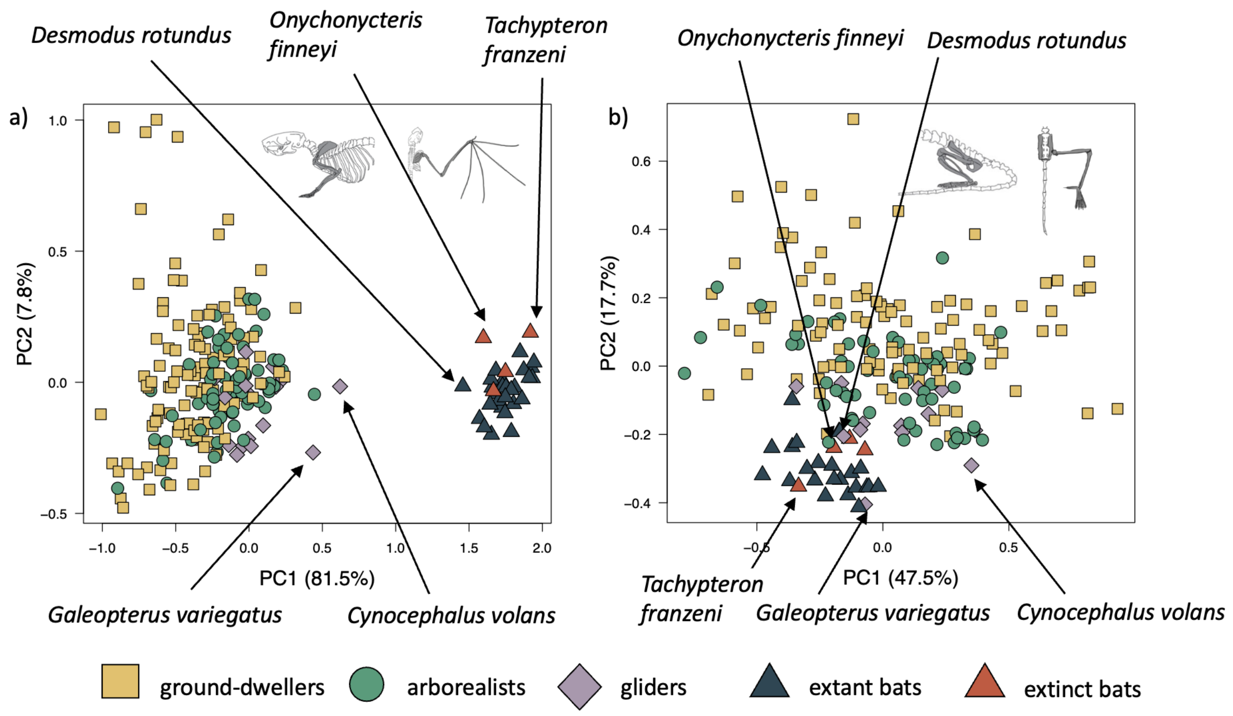

Figure S1: PCA with fossil and extant species of interest labeled. Morphospaces consisting of PCs 1 and 2 of our extant and extinct species' forelimb dataset for a) forelimbs and b) hind limbs with arrows pointing to the dermopteran species *Galeopterus variegatus* and *Cynocephalus volans*; the common vampire bat *Desmodus rotundus*; the most post-cranially primitive fossil bat, *Onychonycteris finneyi*; and *Tachypteron franzeni*, the only extinct species included in this styd that is hypothesized to be part of an extant bat family. All figure components were created by the authors.

Table S1: Loadings for forelimb and hind limb PCAs with extant mammals and extinct bats. Principal component analysis loadings for our a) 14 forelimb traits and b) 10 hind limb traits (pes were excluded due to insufficient data). The five most extreme loadings are bolded for PC1 and PC2.

	**component loadings**		
**a) forelimb traits**	**PC1**	**PC2**	**PC3**
scapula length (sl)	0.056	0.186	0.079
scapula height (sh)	0.099	0.174	**0.258**
humerus length (hl)	**0.212**	0.046	-0.076
humerus shaft width (hsw)	0.088	**0.342**	-0.186
humerus proximal width (hpw)	0.085	0.259	0.000
humerus distal width (hdw)	0.045	**0.344**	**-0.273**
ulna length (ul)	0.256	0.021	-0.089
ulna olecranon length (uol)	-0.066	**0.394**	**0.329**
radius length (rl)	**0.300**	-0.064	-0.115
3rd metacarpal length (mcl)	**0.518**	-0.135	**0.703**
3rd metacarpal width (mcw)	0.068	**0.452**	0.142
3rd proximal phalanx length (ppl)	**0.447**	-0.118	-0.183
3rd proximal phalanx width (ppw)	0.004	**0.483**	-0.017
3rd intermediate phalanx length (ipl)	**0.540**	0.044	**-0.367**
**proportion explained (%)**	81.5	7.8	2.9

**b) hind limb traits**	**PC1**	**PC2**	**PC3**
pelvis length (pel)	0.188	0.189	0.262
ilium length (il)	0.151	0.083	**0.341**
ischium length (isl)	0.250	**0.357**	0.180

femur length (fl)	0.171	-0.25	**0.331**
femur shaft width (fsw)	0.131	0.184	0.182
femur distal width (fdw)	0.141	0.164	0.124
tibia length (tl)	0.272	-0.257	0.239
tibia proximal width (tpw)	0.151	0.141	0.091
tibia midshaft width (tmw)	0.235	0.134	0.118
fibula length (fbl)	**0.301**	-0.204	**0.299**
3rd metatarsal length (mtl)	**0.475**	0.088	-0.211
3rd metatarsal width (mtw)	**0.335**	**0.282**	**-0.448**
3rd pedal proximal phalanx length (ppxl)	**0.310**	**-0.378**	-0.074
3rd pedal proximal phalanx width (ppxw)	0.218	**0.295**	-0.273
3rd intermediate pedal phalanx length (ipxl)	**0.297**	**-0.494**	**-0.362**
**proportion explained (%)**	47.5	17.8	11.3

Table S2: Statistical results for OUM model fitting analyses on the extant-only dataset. Comparisons of the best-fitting OUM models (bolded) using AICc scores to assess fit and AICcW weights to asses relative model support for a) 14 forelimb traits and b) 15 hind limb traits. All models were fit using functions in the *OUwie R* package. Trait abbreviations are explained in table S1.

a. forelimb

sl	fit	delta	w
BM1	-382.99	134.24	0.00
OU1	-501.85	15.39	0.00
OUM_{loc3a}	-510.78	6.45	0.02
**OUM_{loc3b}**	**-517.24**	**0.00**	**0.49**
OUM_{loc3c}	-516.17	1.07	0.28
OUM_{loc4}	-515.57	1.67	0.21

sh	fit	delta	w
BM1	-304.60	92.15	0.00
OU1	-368.87	27.88	0.00
OUM_{loc3a}	-376.88	19.87	0.00
OUM_{loc3b}	-394.81	1.95	0.19
OUM_{loc3c}	-395.61	1.14	0.29
**OUM_{loc4}**	**-396.75**	**0.00**	**0.51**

hl	fit	delta	w
BM1	-418.82	89.49	0.00
OU1	-430.16	78.15	0.00
OUM_{loc3a}	-503.40	4.91	0.08
OUM_{loc3b}	-493.66	14.66	0.00
OUM_{loc3c}	-491.74	16.57	0.00
**OUM_{loc4}**	**-508.31**	**0.00**	**0.92**

hdw	fit	delta	w
BM1	-395.19	43.43	0.00
OU1	-435.24	3.38	0.07
OUM_{loc3a}	-438.61	0.01	0.36
**OUM_{loc3b}**	**-438.62**	**0.00**	**0.36**
OUM_{loc3c}	-434.91	3.71	0.06
OUM_{loc4}	-436.91	1.71	0.15

hsw	fit	delta	w
BM1	-350.21	82.77	0.00
OU1	-410.29	22.69	0.00
OUM_{loc3a}	-426.30	6.69	0.02
**OUM_{loc3b}**	**-432.98**	**0.00**	**0.65**
OUM_{loc3c}	-424.81	8.17	0.01
OUM_{loc4}	-431.55	1.44	0.32

hpw	fit	delta	w
BM1	-451.41	94.80	0.00
OU1	-511.63	34.59	0.00
OUM_{loc3a}	-527.98	18.23	0.00
**OUM_{loc3b}**	**-546.21**	**0.00**	**0.40**
OUM_{loc3c}	-545.50	0.72	0.28
OUM_{loc4}	-545.77	0.44	0.32

rl	fit	delta	w
BM1	-369.45	85.45	0.00
OU1	-368.87	86.03	0.00
OUM_{loc3a}	-442.25	12.66	0.00
OUM_{loc3b}	-437.74	17.17	0.00
OUM_{loc3c}	-441.09	13.81	0.00
**OUM_{loc4}**	**-454.91**	**0.00**	**1.00**

ul	fit	delta	w
BM1	-410.89	113.68	0.00
OU1	-414.76	109.80	0.00
OUM_{loc3a}	-498.02	26.54	0.00
OUM_{loc3b}	-506.08	18.49	0.00
OUM_{loc3c}	-509.29	15.28	0.00
**OUM_{loc4}**	**-524.57**	**0.00**	**1.00**

uol	fit	delta	w
BM1	-272.40	73.47	0.00
OU1	-317.22	28.65	0.00
**OUM_{loc3a}**	**-345.87**	**0.00**	**0.58**
OUM_{loc3b}	-342.30	3.57	0.10
OUM_{loc3c}	-331.90	13.97	0.00
OUM_{loc4}	-344.71	1.16	0.32

mcl	fit	delta	w
BM1	-307.39	55.35	0.00

mcw	fit	delta	w
BM1	-313.12	40.58	0.00

ppl	fit	delta	w
BM1	-308.03	81.41	0.00

OU1	-305.34	57.40	0.00
OUM$_{loc3a}$	-333.09	29.64	0.00
OUM$_{loc3b}$	-359.99	2.75	0.16
**OUM$_{loc3c}$**	**-362.74**	**0.00**	**0.63**
OUM$_{loc4}$	-360.58	2.16	0.21

OU1	-351.03	2.67	0.11
OUM$_{loc3a}$	-348.07	5.64	0.03
**OUM$_{loc3b}$**	**-353.70**	**0.00**	**0.44**
OUM$_{loc3c}$	-352.06	1.65	0.19
OUM$_{loc4}$	-352.42	1.29	0.23

OU1	-306.13	83.31	0.00
OUM$_{loc3a}$	-366.98	22.45	0.00
OUM$_{loc3b}$	-386.09	3.35	0.16
OUM$_{loc3c}$	-367.24	22.20	0.00
**OUM$_{loc4}$**	**-389.44**	**0.00**	**0.84**

ppw	fit	delta	w
BM1	-290.73	53.24	0.00
**OU1**	**-343.97**	**0.00**	**0.51**
OUM$_{loc3a}$	-340.07	3.90	0.07
OUM$_{loc3b}$	-342.91	1.05	0.30
OUM$_{loc3c}$	-339.40	4.56	0.05
OUM$_{loc4}$	-339.78	4.18	0.06

ipl	fit	delta	w
BM1	-221.03	111.43	0.00
OU1	-219.46	113.00	0.00
OUM$_{loc3a}$	-296.07	36.39	0.00
OUM$_{loc3b}$	-323.22	9.24	0.01
OUM$_{loc3c}$	-317.39	15.07	0.00
**OUM$_{loc4}$**	**-332.46**	**0.00**	**0.99**

b. hind limb

fbl	fit	delta	w
BM1	-314.06	34.02	0.00
OU1	-341.05	7.03	0.02
OUM$_{loc3a}$	-340.55	7.53	0.01
OUM$_{loc3b}$	-344.93	3.15	0.13
OUM$_{loc3c}$	-346.23	1.86	0.24
**OUM$_{loc4}$**	**-348.08**	**0.00**	**0.60**

fdw	fit	delta	w
BM1	-459.10	93.67	0.00
OU1	-533.90	18.87	0.00
OUM$_{loc3a}$	-549.84	2.93	0.10
OUM$_{loc3b}$	-551.60	1.17	0.23
**OUM$_{loc3c}$**	**-552.77**	**0.00**	**0.42**
OUM$_{loc4}$	-551.80	0.97	0.26

fl	fit	delta	w
BM1	-390.02	37.29	0.00
OU1	-408.25	19.06	0.00
OUM$_{loc3a}$	-425.56	1.75	0.28
OUM$_{loc3b}$	-421.54	5.77	0.04
OUM$_{loc3c}$	-416.18	11.13	0.00
**OUM$_{loc4}$**	**-427.31**	**0.00**	**0.68**

fsw	fit	delta	w
BM1	-413.39	83.94	0.00
OU1	-488.60	8.74	0.01
**OUM$_{loc3a}$**	**-497.33**	**0.00**	**0.52**
OUM$_{loc3b}$	-492.90	4.43	0.06
OUM$_{loc3c}$	-495.90	1.44	0.26
OUM$_{loc4}$	-494.89	2.44	0.16

il	fit	delta	w
BM1	-425.75	51.39	0.00
OU1	-468.39	8.75	0.01
OUM$_{loc3a}$	-471.22	5.93	0.03
**OUM$_{loc3b}$**	**-477.14**	**0.00**	**0.52**
OUM$_{loc3c}$	-473.59	3.55	0.09
OUM$_{loc4}$	-476.37	0.77	0.36

ipxl	fit	delta	w
BM1	-95.48	97.23	0.00
OU1	-182.86	9.84	0.00
OUM$_{loc3a}$	-191.41	1.30	0.27
**OUM$_{loc3b}$**	**-192.70**	**0.00**	**0.51**
OUM$_{loc3c}$	-186.99	5.72	0.03
OUM$_{loc4}$	-190.67	2.04	0.19

isl	fit	delta	w
BM1	-357.04	60.19	0.00
OU1	-392.70	24.54	0.00
OUM$_{loc3a}$	-412.19	5.04	0.06
**OUM$_{loc3b}$**	**-417.23**	**0.00**	**0.71**
OUM$_{loc3c}$	-411.63	5.60	0.04
OUM$_{loc4}$	-414.57	2.67	0.19

mtl	fit	delta	w
BM1	-232.90	17.42	0.00
OU1	-243.85	6.47	0.02
OUM$_{loc3a}$	-249.63	0.69	0.31
**OUM$_{loc3b}$**	**-250.32**	**0.00**	**0.44**
OUM$_{loc3c}$	-246.06	4.26	0.05
OUM$_{loc4}$	-248.60	1.72	0.18

mtw	fit	delta	w
BM1	-87.72	144.14	0.00
OU1	-215.33	16.52	0.00
**OUM$_{loc3a}$**	**-231.86**	**0.00**	**0.50**
OUM$_{loc3b}$	-230.38	1.47	0.24
OUM$_{loc3c}$	-226.54	5.31	0.04
OUM$_{loc4}$	-230.20	1.65	0.22

pel	fit	delta	w
BM1	-437.41	62.83	0.00
OU1	-483.10	17.14	0.00
OUM$_{loc3a}$	-491.45	8.79	0.01
OUM$_{loc3b}$	-499.65	0.59	0.35
**OUM$_{loc3c}$**	**-500.24**	**0.00**	**0.48**
OUM$_{loc4}$	-498.11	2.13	0.16

ppxl	fit	delta	w
BM1	-257.05	21.01	0.00
**OU1**	**-278.06**	**0.00**	**0.42**
OUM$_{loc3a}$	-276.13	1.93	0.16
OUM$_{loc3b}$	-276.53	1.53	0.20
OUM$_{loc3c}$	-274.98	3.08	0.09
OUM$_{loc4}$	-275.79	2.27	0.13

ppxw	fit	delta	w
BM1	-161.50	131.90	0.00
OU1	-288.94	4.46	0.04
**OUM$_{loc3a}$**	**-293.40**	**0.00**	**0.36**
OUM$_{loc3b}$	-293.32	0.08	0.35
OUM$_{loc3c}$	-291.10	2.30	0.11
OUM$_{loc4}$	-291.43	1.97	0.14

tl	fit	delta	w
BM1	-364.70	22.93	0.00
OU1	-382.77	4.86	0.03
OUM$_{loc3a}$	-387.32	0.31	0.28
OUM$_{loc3b}$	-384.09	3.54	0.06
OUM$_{loc3c}$	-387.55	0.09	0.31
**OUM$_{loc4}$**	**-387.63**	**0.00**	**0.33**

tmw	fit	delta	w
BM1	-309.05	78.44	0.00
**OU1**	**-387.49**	**0.00**	**0.46**
OUM$_{loc3a}$	-386.13	1.36	0.23
OUM$_{loc3b}$	-385.31	2.18	0.16
OUM$_{loc3c}$	-384.33	3.16	0.09
OUM$_{loc4}$	-383.24	4.26	0.05

tpw	fit	delta	w
BM1	-352.62	108.62	0.00
OU1	-449.69	11.56	0.00
OUM$_{loc3a}$	-459.33	1.92	0.18
OUM$_{loc3b}$	-459.65	1.60	0.22
**OUM$_{loc3c}$**	**-461.25**	**0.00**	**0.48**
OUM$_{loc4}$	-458.45	2.79	0.12

Table S3: Optima and bootstrapping results for best-supported OUM model. We bootstrapped traits with supported OUM models and excluded traits with supported OU1 (proximal phalanx width, tibia midshaft width, pedal proximal phalanx length) for the a) forelimb and b) hind limbs. Trait abbreviations are explained in Figure S1 and table S1.

a. forelimb mean θ values and 95% confidence intervals for 13 traits (1 excluded) for the best supported model.

**sl**	**OUM_{loc3b} optima**	**L95**	**U95**
ground	0.58	0.56	0.60
arb+glide	0.59	0.57	0.61
fly	0.67	0.64	0.71

**sh**	**OUM_{loc4} optima**	**L95**	**U95**
ground	0.30	0.28	0.33
arb.	0.32	0.28	0.35
glide	0.24	0.15	0.33
fly	0.51	0.46	0.57

**hl**	**OUM_{loc4} optima**	**L95**	**U95**
ground	0.63	0.61	0.66
arb.	0.74	0.70	0.77
glide	0.93	0.83	1.03
fly	1.10	1.03	1.16

**hdw**	**OUM_{loc3b} optima**	**L95**	**U95**
ground	0.09	0.06	0.13
arb+glide	0.15	0.10	0.20
fly	0.22	0.14	0.31

**hsw**	**OUM_{loc3b} optima**	**L95**	**U95**
ground	-0.40	-0.42	-0.38
arb+glide	-0.34	-0.37	-0.32
fly	-0.23	-0.27	-0.18

**hpw**	**OUM_{loc3b} optima**	**L95**	**U95**
ground	0.01	-0.01	0.03
arb+glide	0.01	-0.01	0.03
fly	0.18	0.14	0.22

**rl**	**OUM_{loc4} optima**	**L95**	**U95**
ground	0.60	0.57	0.63
arb.	0.73	0.68	0.77
glide	1.00	0.87	1.13
fly	1.33	1.24	1.41

**ul**	**OUM_{loc4} optima**	**L95**	**U95**
ground	0.71	0.69	0.73
arb.	0.79	0.76	0.82
glide	0.97	0.89	1.05
fly	1.29	1.24	1.33

**uol**	**OUM_{loc3a} optima**	**L95**	**U95**
ground	-0.09	-0.11	-0.06
arb.	-0.18	-0.22	-0.15
glide+fly	-0.28	-0.33	-0.23

**mcl**	**OUM_{loc3c} optima**	**L95**	**U95**
ground+arb	0.04	-0.08	0.15
glide	0.24	-0.31	0.78
fly	2.41	1.50	3.68

mcw	OUM_{loc3b} optima	L95	U95
ground	-0.71	-0.75	-0.68
arb+glide	-0.77	-0.83	-0.71
fly	-0.59	-0.69	-0.49

ppl	OUM_{loc4} optima	L95	U95
ground	-0.22	-0.28	-0.17
arb.	0.06	-0.05	0.15
glide	0.28	0.01	0.52
**fly**	1.14	0.83	1.39

ipl	OUM_{loc4} optima	L95	U95
ground	-0.32	-0.37	-0.28
arb.	-0.13	-0.21	-0.06
glide	0.17	-0.05	0.38
**fly**	1.06	0.85	1.25

b. hind limb mean θ values and 95% confidence intervals for 13 traits (2 excluded) for the best supported model.

fbl	OUM_{loc4} optima	L95	U95
ground	0.76	0.72	0.80
arb.	0.85	0.78	0.91
glide	1.05	0.85	1.23
fly	0.72	0.61	0.82

fl	OUM_{loc4} optima	L95	U95
ground	0.72	0.69	0.75
arb.	0.85	0.79	0.89
glide	1.01	0.87	1.14
fly	0.86	0.78	0.94

il	OUM_{loc3b} optima	L95	U95
ground	0.54	0.51	0.56
arb+glide	0.59	0.56	0.62
fly	0.45	0.39	0.51

fdw	OUM_{loc3c} optima	L95	U95
ground+arb	0.06	0.05	0.07
glide	0.03	-0.02	0.08
fly	-0.05	-0.09	-0.01

fsw	OUM_{loc3a} optima	L95	U95
ground	-0.29	-0.31	-0.27
arb.	-0.29	-0.31	-0.26
glide+fly	-0.36	-0.40	-0.33

ipxl	OUM_{loc3b} optima	L95	U95
ground	-0.20	-0.24	-0.17
arb+glide	-0.12	-0.16	-0.08
fly	-0.05	-0.13	0.03

mtl	OUM_{loc3b} optima	L95	U95
ground	0.37	0.27	0.48

**isl**	**OUM_{loc3b} optima**	**L95**	**U95**
ground	0.37	0.35	0.40
arb+glide	0.31	0.28	0.35
fly	0.13	0.07	0.20

**mtw**	**OUM_{loc3a} optima**	**L95**	**U95**
ground	-0.64	-0.67	-0.61
arb.	-0.71	-0.75	-0.67
glide+fly	-0.79	-0.84	-0.73

**ppxw**	**OUM_{loc3a} optima**	**L95**	**U95**
ground	-0.69	-0.72	-0.67
arb.	-0.73	-0.77	-0.70
glide+fly	-0.77	-0.82	-0.72

**tpw**	**OUM_{loc3c} optima**	**L95**	**U95**
ground+arb	0.05	0.03	0.06
glide	0.01	-0.04	0.06
fly	-0.04	-0.08	0.00

arb+glide	0.17	0.05	0.32
fly	-0.03	-0.28	0.25

**pel**	**OUM_{loc3c} optima**	**L95**	**U95**
ground+arb	0.77	0.76	0.79
glide	0.79	0.71	0.87
fly	0.62	0.57	0.67

**tl**	**OUM_{loc4} optima**	**L95**	**U95**
ground	0.79	0.74	0.83
arb.	0.86	0.78	0.93
glide	1.09	0.86	1.28
fly	0.89	0.76	1.00

Table S4: Statistical results for model fitting analyses on the extant+extinct dataset. Comparisons of the best-fitting OUM models (bolded) using AICc scores to asses fit and AICcW weights to asses relative model support for a) 14 forelimb traits and b) 15 hind limb traits. We fit all models using functions in the *OUwie R* package. Trait abbreviations are explained in table S1.

a. Forelimb

**hdw**	**fit**	**delta**	**w**
BM1	-414.31	36.66	0
OU1	-448.77	2.2	0.14
OUM$_{loc3a}$	-448.46	2.51	0.12
**OUM$_{loc3b}$**	**-450.97**	**0**	**0.41**
OUM$_{loc3c}$	-448.86	2.11	0.14
OUM$_{loc4}$	-449.54	1.43	0.2

**hl**	**fit**	**delta**	**w**
BM1	-414.17	87.35	0
OU1	-435.55	65.97	0
OUM$_{loc3a}$	-489.84	11.68	0
OUM$_{loc3b}$	-489.51	12.01	0
OUM$_{loc3c}$	-490.29	11.23	0
**OUM$_{loc4}$**	**-501.52**	**0**	**0.99**

**hpw**	**fit**	**delta**	**w**
BM1	-469.58	89.9	0
OU1	-523.52	35.96	0
**OUM$_{loc3a}$**	**-559.48**	**0**	**0.35**
OUM$_{loc3b}$	-559	0.48	0.27
OUM$_{loc3c}$	-558.82	0.66	0.25
OUM$_{loc4}$	-557.53	1.95	0.13

**hsw**	**fit**	**delta**	**w**
BM1	-364	72.66	0
OU1	-416.5	20.17	0
OUM$_{loc3a}$	-430.17	6.5	0.03
**OUM$_{loc3b}$**	**-436.67**	**0**	**0.66**
OUM$_{loc3c}$	-429.92	6.74	0.02
OUM$_{loc4}$	-435.04	1.63	0.29

**ipl**	**fit**	**delta**	**w**
BM1	-182.49	106.48	0
OU1	-191.73	97.24	0
OUM$_{loc3a}$	-280.35	8.62	0.01
OUM$_{loc3b}$	-283.97	5	0.07
OUM$_{loc3c}$	-281.14	7.83	0.02
**OUM$_{loc4}$**	**-288.97**	**0**	**0.9**

**mcl**	**fit**	**delta**	**w**
BM1	-292.42	53.72	0
OU1	-295.49	50.65	0
OUM$_{loc3a}$	-340.94	5.2	0.04
**OUM$_{loc3b}$**	**-346.14**	**0**	**0.58**
OUM$_{loc3c}$	-345.19	0.95	0.36
OUM$_{loc4}$	-338.44	7.7	0.01

**mcw**	**fit**	**delta**	**w**
BM1	-319.17	40.19	0
OU1	-356.63	2.74	0.1
OUM$_{loc3a}$	-357.84	1.52	0.19
**OUM$_{loc3b}$**	**-359.36**	**0**	**0.4**
OUM$_{loc3c}$	-357.69	1.67	0.17
OUM$_{loc4}$	-357.2	2.16	0.14

**ppl**	**fit**	**delta**	**w**
BM1	-299.33	74.17	0
OU1	-300.18	73.32	0
OUM$_{loc3a}$	-351.81	21.69	0
OUM$_{loc3b}$	-372.28	1.22	0.35
OUM$_{loc3c}$	-351.11	22.39	0
**OUM$_{loc4}$**	**-373.5**	**0**	**0.65**

**ppw**	**fit**	**delta**	**w**
BM1	-305.14	47.54	0
**OU1**	**-352.68**	**0**	**0.55**
OUM$_{loc3a}$	-348.69	3.99	0.08
OUM$_{loc3b}$	-350.86	1.82	0.22
OUM$_{loc3c}$	-349.03	3.65	0.09
OUM$_{loc4}$	-348.13	4.55	0.06

**rl**	fit	delta	w
BM1	-357.08	83.62	0
OU1	-365.86	74.84	0
OUM$_{loc3a}$	-427.88	12.82	0
OUM$_{loc3b}$	-427	13.7	0

sh	fit	delta	w
BM1	-315.44	89.03	0
OU1	-376.83	27.64	0
**OUM$_{loc3a}$**	**-404.47**	**0**	**0.42**
OUM$_{loc3b}$	-401.68	2.79	0.1

sl	fit	delta	w
BM1	-393.8	133.47	0
OU1	-506.44	20.83	0
OUM$_{loc3a}$	-526.78	0.49	0.26
**OUM$_{loc3b}$**	**-527.27**	**0**	**0.33**

OUM$_{loc3c}$	-429.04	11.66	0	OUM$_{loc3c}$	-403.43	1.04	0.25	OUM$_{loc3c}$	-527.05	0.22	0.3
**OUM$_{loc4}$**	**-440.7**	**0**	**0.99**	OUM$_{loc4}$	-403.29	1.19	0.23	OUM$_{loc4}$	-524.93	2.34	0.1

**ul**	fit	delta	w	**uol**	**fit**	**delta**	**w**
BM1	-401.44	124.63	0	BM1	-264.02	82.47	0
OU1	-409.77	116.3	0	OU1	-314.8	31.69	0
OUM$_{loc3a}$	-509.25	16.82	0	OUM$_{loc3a}$	-334.21	12.28	0
OUM$_{loc3b}$	-498.37	27.7	0	OUM$_{loc3b}$	-345.14	1.35	0.34
OUM$_{loc3c}$	-502.25	23.82	0	OUM$_{loc3c}$	-333.88	12.6	0
**OUM$_{loc4}$**	**-526.07**	**0**	**1**	**OUM$_{loc4}$**	**-346.49**	**0**	**0.66**

b. Hind limb

**fbl**	**fit**	**delta**	**w**	**fdw**	**fit**	**delta**	**w**	**fl**	**fit**	**delta**	**w**
BM1	-336.31	24.84	0	BM1	-464.14	95.9	0	BM1	-399.43	34.99	0
OU1	-356.4	4.76	0.05	OU1	-536.38	23.66	0	OU1	-417.78	16.64	0
OUM$_{loc3a}$	-355.13	6.03	0.03	OUM$_{loc3a}$	-556.29	3.75	0.06	OUM$_{loc3a}$	-431.38	3.03	0.17
OUM$_{loc3b}$	-357.8	3.35	0.11	OUM$_{loc3b}$	-559.52	0.52	0.29	OUM$_{loc3b}$	-429.49	4.93	0.06
OUM$_{loc3c}$	-359.53	1.62	0.25	**OUM$_{loc3c}$**	**-560.04**	**0**	**0.37**	OUM$_{loc3c}$	-423.86	10.56	0
**OUM$_{loc4}$**	**-361.15**	**0**	**0.56**	OUM$_{loc4}$	-559.46	0.58	0.28	**OUM$_{loc4}$**	**-434.41**	**0**	**0.76**

**fsw**	**fit**	**delta**	**w**	**il**	**fit**	**delta**	**w**	**ipxl**	**fit**	**delta**	**w**
BM1	-419.08	80.97	0	BM1	-428.09	50.06	0	BM1	-99.84	97.13	0
OU1	-492.23	7.83	0.01	OU1	-471.26	6.89	0.02	OU1	-186.3	10.67	0
**OUM$_{loc3a}$**	**-500.05**	**0**	**0.42**	OUM$_{loc3a}$	-473.08	5.06	0.04	OUM$_{loc3a}$	-195.94	1.03	0.28
OUM$_{loc3b}$	-496.49	3.57	0.07	**OUM$_{loc3b}$**	**-478.15**	**0**	**0.54**	**OUM$_{loc3b}$**	**-196.97**	**0**	**0.47**
OUM$_{loc3c}$	-499.82	0.24	0.37	OUM$_{loc3c}$	-474.07	4.08	0.07	OUM$_{loc3c}$	-191.71	5.26	0.03
OUM$_{loc4}$	-497.82	2.23	0.14	OUM$_{loc4}$	-477.21	0.94	0.34	OUM$_{loc4}$	-195.29	1.68	0.2

**isl**	**fit**	**delta**	**w**	**mtl**	**fit**	**delta**	**w**	**pel**	**fit**	**delta**	**w**
BM1	-362.54	59.58	0	BM1	-234.47	15.71	0	BM1	-446.28	59.11	0
OU1	-393.96	28.16	0	OU1	-244.72	5.46	0.03	OU1	-489.28	16.11	0
OUM$_{loc3a}$	-416.29	5.82	0.04	OUM$_{loc3a}$	-249.61	0.57	0.35	OUM$_{loc3a}$	-498.44	6.94	0.01
**OUM$_{loc3b}$**	**-422.12**	**0**	**0.76**	**OUM$_{loc3b}$**	**-250.18**	**0**	**0.46**	**OUM$_{loc3b}$**	**-505.38**	**0**	**0.43**
OUM$_{loc3c}$	-415.33	6.79	0.03	OUM$_{loc3c}$	-244.74	5.45	0.03	OUM$_{loc3c}$	-505.19	0.2	0.39
OUM$_{loc4}$	-419.17	2.94	0.17	OUM$_{loc4}$	-247.6	2.58	0.13	OUM$_{loc4}$	-503.58	1.81	0.17

**tl**	**fit**	**delta**	**w**
BM1	-370.43	22.64	0
OU1	-388.18	4.9	0.04
OUM_{loc3a}	-391.47	1.6	0.19
OUM_{loc3b}	-389.18	3.9	0.06
OUM_{loc3c}	-392.24	0.83	0.28
**OUM_{loc4}**	**-393.07**	**0**	**0.43**

**tmw**	**fit**	**delta**	**w**
BM1	-300.03	85.01	0
**OU1**	**-385.04**	**0**	**0.44**
OUM_{loc3a}	-383.38	1.66	0.19
OUM_{loc3b}	-383.46	1.58	0.2
OUM_{loc3c}	-382	3.04	0.1
OUM_{loc4}	-381.33	3.71	0.07

**tpw**	**fit**	**delta**	**w**
BM1	-358.46	107.88	0
OU1	-450.83	15.52	0
OUM_{loc3a}	-464.22	2.13	0.16
OUM_{loc3b}	-464.86	1.49	0.22
**OUM_{loc3c}**	**-466.35**	**0**	**0.46**
OUM_{loc4}	-464.32	2.03	0.17

**ppxw**	**fit**	**delta**	**w**
BM1	-153.48	136.46	0
OU1	-283.09	6.86	0.01
**OUM_{loc3a}**	**-289.94**	**0**	**0.39**
OUM_{loc3b}	-289.66	0.28	0.34
OUM_{loc3c}	-287.42	2.52	0.11
OUM_{loc4}	-288.12	1.82	0.16

**ppxl**	**fit**	**delta**	**w**
BM1	-253.68	22.28	0
**OU1**	**-275.97**	**0**	**0.44**
OUM_{loc3a}	-274.31	1.66	0.19
OUM_{loc3b}	-274.44	1.53	0.2
OUM_{loc3c}	-272.48	3.48	0.08
OUM_{loc4}	-272.94	3.02	0.1

**mtw**	**fit**	**delta**	**w**
BM1	-72.59	160.39	0
OU1	-212.33	20.65	0
**OUM_{loc3a}**	**-232.98**	**0**	**0.58**
OUM_{loc3b}	-230.79	2.19	0.19
OUM_{loc3c}	-226.85	6.13	0.03
OUM_{loc4}	-230.89	2.09	0.2

Table S5: Optima values for the best-fitting OUM model results for extant+extinct dataset. Bootstrapping did not perform well on this dataset, so here we include only the model optima values with upper and lower 95% confidence intervals for a) forelimb traits and b) hind limb traits. We excluded traits only supported by an OU1 model (proximal phalanx width and pedal proximal phalanx length). Trait abbreviations are explained in Figure S1 and table S1.

a. forelimb

sl	OUMloc3b optima	L95	U95
ground	0.58	0.58	0.58
arb+glide	0.59	0.59	0.59
fly	0.68	0.68	0.68

sh	OUMloc3a optima	L95	U95
ground	0.31	0.31	0.31
arb.	0.24	0.23	0.26
glide+fly	0.50	0.50	0.51

uol	OUMloc4 optima	L95	U95
ground	-0.09	-0.09	-0.08
arb.	-0.18	-0.19	-0.17
glide	-0.27	-0.31	-0.22
fly	-0.28	-0.28	-0.27

hdw	OUMloc3b optima	L95	U95
ground	0.09	0.07	0.11
arb. & glide	0.15	0.12	0.17
fly	0.21	0.21	0.21

hsw	OUMloc3b optima	L95	U95
ground	-0.40	-0.41	-0.40
arb. & glide	-0.35	-0.35	-0.33
fly	-0.21	-0.21	-0.21

hpw	OUMloc3a optima	L95	U95
ground	0.01	0.01	0.01
arb.	0.00	-0.01	0.00
glide+fly	0.19	0.19	0.19

rl	OUMloc4 optima	L95	U95
ground	0.61	0.60	0.62
arb.	0.72	0.70	0.73
glide	0.93	0.87	1.01
fly	1.29	1.29	1.31

ul	OUMloc4 optima	L95	U95
ground	0.71	0.71	0.72
arb.	0.79	0.78	0.80
glide	0.94	0.91	0.97
fly	1.29	1.29	1.29

mcl	OUMloc3b optima	L95	U95
ground	-0.04	-0.10	0.01
arb. & glide	0.03	-0.05	0.08
fly	1.44	1.37	1.48

mcw	OUMloc3b optima	L95	U95
ground	-0.72	-0.73	-0.70
arb. & glide	-0.77	-0.80	-0.74
fly	-0.60	-0.61	-0.60

hl	OUMloc4 optima	L95	U95
ground	0.64	0.63	0.64
arb.	0.73	0.72	0.75
glide	0.93	0.86	0.99
fly	1.09	1.09	1.10

ppl	OUMloc4 optima	L95	U95
ground	-0.23	-0.28	-0.19
arb.	0.04	-0.02	0.10
glide	0.24	0.15	0.31
fly	0.92	0.89	0.95

ipl	OUMloc4 optima	L95	U95
ground	-0.32	-0.34	-0.30
arb.	-0.15	-0.19	-0.12
glide	0.13	0.02	0.22
fly	0.95	0.93	0.97

b. hind limb

pel	OUMloc4b optima	L95	U95
ground	0.77	0.77	0.78
arb+glide	0.78	0.77	0.79
fly	0.63	0.63	0.63

il	OUMloc3b optima	L95	U95
ground	0.54	0.53	0.55
arb+glide	0.59	0.57	0.60
fly	0.46	0.46	0.46

isl	OUMloc3b optima	L95	U95
ground	0.38	0.37	0.38
arb+glide	0.31	0.29	0.32
fly	0.13	0.13	0.13

fsw	OUMloc3a optima	L95	U95
ground	-0.29	-0.29	-0.29
arb.	-0.36	-0.37	-0.35
glide+fly	-0.35	-0.35	-0.35

fl	OUMloc4 optima	L95	U95
ground	0.72	0.71	0.73
arb.	0.85	0.81	0.88
glide	1.03	0.98	1.12
fly	0.86	0.85	0.86

tl	OUMloc4 optima	L95	U95
ground	0.79	0.77	0.80
arb.	0.86	0.82	0.90
glide	1.12	1.04	1.23
fly	0.88	0.87	0.88

fbl	OUMloc4 optima	L95	U95
ground	0.76	0.74	0.77
arb.	0.84	0.80	0.88
glide	1.10	1.03	1.21
fly	0.71	0.70	0.72

tpw	OUMloc3c optima	L95	U95
ground+arb	0.05	0.05	0.05
glide	0.01	0.00	0.02
fly	-0.05	-0.05	-0.05

mtl	OUMloc3b optima	L95	U95
ground	0.39	0.36	0.43
arb+glide	0.19	0.12	0.25
fly	0.09	0.08	0.10

mtw	OUMloc3a optima	L95	U95
ground	-0.64	-0.65	-0.64
arb.	-0.71	-0.72	-0.70
glide+fly	-0.80	-0.80	-0.79

ipxl	optima	L95	U95
ground	-0.20	-0.21	-0.20
arb+glide	-0.12	-0.13	-0.12
fly	-0.05	-0.05	-0.05

ppxw	OUMloc3a optima	L95	U95
ground	-0.70	-0.70	-0.69
arb.	-0.73	-0.74	-0.73
glide+fly	-0.79	-0.79	-0.78

fdw	OUMloc3c optima	L95	U95
**ground+arb**	0.06	0.06	0.06
**glide**	0.03	0.01	0.04
**fly**	-0.05	-0.05	-0.05

Table S6. Fits of multivariate evolutionary models to morphological data of extant+extinct species. A) MvMORPH models were fit to PC1–PC3 scores of a principal component analysis PCA of 29 skeletal traits. B) Optima were extracted from the model, but were physically impossible for the forelimb models so cannot be properly analyzed.

a)

forelimb	fit	delta	w
mvBM1	-514.32	239.51	0.00
mvOU1	-561.39	192.44	0.00
$mvOUM_{loc3a}$	-722.22	31.60	0.00
$mvOUM_{loc3b}$	-730.87	22.95	0.00
$mvOUM_{loc3c}$	-712.38	41.45	0.00
**$mvOUM_{loc4}$**	**-753.83**	**0.00**	**1.00**

hind limb	fit	delta	w
mvBM1	-241.16	212.95	0.00
mvOU1	-400.68	53.43	0.00
$mvOUM_{loc3a}$	-450.88	3.24	0.16
$mvOUM_{loc3b}$	-447.86	6.25	0.04
$mvOUM_{loc3c}$	-435.10	19.01	0.00
**$mvOUM_{loc4}$**	**-454.11**	**0.00**	**0.80**

b) PC1 and PC2 optima are formatted as points (PC1, PC2) that could be superimposed on a PCA.

best supported model	ground-dweller optimum	arborealist optimum	glider optimum	bat optimum
forelimb $mvMORPH_{loc4}$ PC1 value	-118.91	-100.66	-55.78	-6.85
forelimb $mvMORPH_{loc4}$ PC2 value	-256.49	-214.10	-107.61	-24.24
hind limb $mvMORPH_{loc4}$ PC1 value	0.03	0.01	-0.01	-0.20
hind limb $mvMORPH_{loc4}$ PC2 value	0.03	0.00	-0.02	0.20
